# Model of therapeutic intervention in anorexia nervosa of adolescents with depressive behavioral disorders

**DOI:** 10.1192/j.eurpsy.2021.601

**Published:** 2021-08-13

**Authors:** I. Mykhailova, D. Mitelov, T. Matkovska, O. Mayorov

**Affiliations:** 1 Medical, Kharkiv National Medical University, Kharkiv, Ukraine; 2 Psychiatry, State Institution “Institute of the Health Care of Children and Adolescents of NAMS of Ukraine”, Kharkiv, Ukraine; 3 Clinical Informatics And Information Technology In Health Care Management, Kharkiv Medical Academy of Postgraduate Education of the Ministry of Health of Ukraine, Kharkiv, Ukraine

**Keywords:** depression, adolescent, behavioral disorders, anorexia nervosa

## Abstract

**Introduction:**

Anorexia nervosa is often associated with the development of depressive disorders.

**Objectives:**

Skillful dissimulation of the true causes of fasting in adolescents leads to diagnostic errors and delayed adequate therapy.

**Methods:**

The study design includes clinical psychopathological, somatic-neurological, and psychological methods for examination of 54 adolescent girls aged 12-14, with a recurrent depression, and factors determine disorders in alimentary behavior (anorexia nervosa) in teen-agers. The following psychological tests were performed: Children’s Depression Rating Scale Revised, Columbia - Suicide Severity Rating Scale, Mendelevitch - Yakhin Scale to establish a neurotic state.

**Results:**

in all adolescent girls with anorexia nervosa depressive disorders were present in prepuberty. Behavioral syndrome and aggressive vulnerability prevailed in the structure of depression. Cognitive component was represented in the form of unstable type of poor memory and decreased rate of sensorimotor reactions with episodic recurrent attacks of bulimia. Our model of therapeutic intervention included: behavioral intervention, intravenous administration of Cerebrolysin 10,0 with 0,9 % Sodium chloride 200,0 (No.15). Therapeutic neuroplasticity, multimodal effect, and a disease - modifying therapy effects in short terms provide regression of emotional-cognitivity.

**Conclusions:**

In adolescent girls with a recurrent depression anorexia nervosa has specific features that require early differentiation, neurotropic and neurodegenerative therapy.

